# Novel Perspectives in Redox Biology and Pathophysiology of Failing Myocytes: Modulation of the Intramyocardial Redox Milieu for Therapeutic Interventions—A Review Article from the Working Group of Cardiac Cell Biology, Italian Society of Cardiology

**DOI:** 10.1155/2016/6353469

**Published:** 2016-01-05

**Authors:** Alessia Arcaro, Flora Pirozzi, Annalisa Angelini, Cristina Chimenti, Lia Crotti, Carla Giordano, Daniele Mancardi, Daniele Torella, Carlo G. Tocchetti

**Affiliations:** ^1^Department of Medicine and Health Sciences, University of Molise, 86100 Campobasso, Italy; ^2^Department of Translational Medical Sciences, Division of Internal Medicine, Federico II University, 80131 Naples, Italy; ^3^Cardiovascular Pathology Unit, Department of Thoracic, Cardiac and Vascular Sciences, University of Padova, 35121 Padova, Italy; ^4^La Sapienza University and IRCCS L. Spallanzani, 00161 Rome, Italy; ^5^Department of Molecular Medicine, University of Pavia, 27100 Pavia, Italy; ^6^Center for Cardiac Arrhythmias of Genetic Origin, IRCCS Istituto Auxologico Italiano, 20135 Milano, Italy; ^7^Department of Radiological, Oncological and Anatomo-Pathological Sciences, La Sapienza University, 00161 Rome, Italy; ^8^Department of Clinical and Biological Sciences, University of Torino, 10043 Orbassano, Italy; ^9^Molecular and Cellular Cardiology, Department of Medical and Surgical Sciences, Magna Graecia University, 88100 Catanzaro, Italy

## Abstract

The prevalence of heart failure (HF) is still increasing worldwide, with enormous human, social, and economic costs, in spite of huge efforts in understanding pathogenetic mechanisms and in developing effective therapies that have transformed this syndrome into a chronic disease. Myocardial redox imbalance is a hallmark of this syndrome, since excessive reactive oxygen and nitrogen species can behave as signaling molecules in the pathogenesis of hypertrophy and heart failure, leading to dysregulation of cellular calcium handling, of the contractile machinery, of myocardial energetics and metabolism, and of extracellular matrix deposition. Recently, following new interesting advances in understanding myocardial ROS and RNS signaling pathways, new promising therapeutical approaches with antioxidant properties are being developed, keeping in mind that scavenging ROS and RNS* tout court* is detrimental as well, since these molecules also play a role in physiological myocardial homeostasis.

## 1. Introduction

The prevalence of heart failure (HF) is still increasing worldwide, with enormous human, social, and economic costs [1–6], despite huge efforts in understanding pathogenetic mechanisms and in developing effective therapies that have transformed this syndrome into a chronic disease. Recently, following new interesting advances in understanding intracellular signaling pathways that control the main altered processes in the failing heart (such as cellular calcium handling and the contractile machinery, cardiac hypertrophy and dilatation, and myocardial energetics and metabolism), new promising therapeutical approaches are being developed. It is well established that cardiomyocytes of a failing heart are redox imbalanced, and, in this paper, we review and discuss the pathophysiology of HF, keeping in mind that ROS and RNS play an important role as signaling molecules in physiological myocardial homeostasis.

## 2. Heart Failure as a Systemic Disease

The etiology of heart dysfunction is heterogeneous, although individuals with HF have rather common symptoms as fatigue, shortness of breath, and fluid retention. Half of HF patients present with contractile failure and a dilated heart (systolic HF), while other patients have normal systolic function with a preserved ejection fraction (EF) and a nondilated, but often hypertrophied, heart. This latter is also named HF with preserved ejection fraction (HFpEF) [7].

Central to the pathogenesis of systolic HF is decreased left ventricular (LV) contractile function, due to an initial ischaemic insult (e.g., myocardial infarction, MI) or to nonischaemic insult (including genetic and inflammatory cardiomyopathies, hypertension, metabolic diseases, or toxic injury). These insults induce an inexorable series of compensatory responses in the body, including the retention of salt and water by the kidneys, the release of neurohormones, and the activation of intracellular signaling cascades in the heart and vasculature that modify cellular and organ morphology and function. Such responses initially offset reduced cardiac performance but then become part of the disease process, increasing organ failure and worsening clinical prognosis [1–7].

## 3. Neurohumoral Adaptations

When cardiac contractile dysfunction is established, the body responds by increasing release of sympathetic neurotransmitters, adrenaline and noradrenaline, and neurohormones, including angiotensin II (ATII), endothelin, and natriuretic peptides. These events contribute to maintaining cardiac output, increasing rate and intensity of heart contraction, and fluid retention. Such chronic stimulation becomes adverse and worsens prognosis of heart failure [8]. Indeed, current HF therapies mainly rely on antagonizing such neurohumoral activated pathways with *β*-adrenergic and angiotensin receptor blockade and angiotensin converting enzyme (ACE) inhibition and on hemodynamic control with nitrates and diuretics, with the net effect of producing vasodilation and lowering blood pressure, therefore unloading the heart [1–7]. Therapies based on blockade of *β*-adrenergic receptors (*β*-ARs), inhibition of angiotensin converting enzyme, blockade of the angiotensin II receptor AT1, and blockade of aldosterone receptor improved survival and symptoms in heart failure patients [1, 7, 9–11].

## 4. Energetic Breakdown in Heart Failure

The heart is an organ with limited capacity for storing energy. Thereby, to supply its high and constant workload, it needs substrates produced quickly and efficiently, mainly from circulating fatty acids (FA) rather than from glucose. A failing heart enters a state of inefficiency and of energy starvation, mainly due to a compromised regulation of energy metabolism, a reduced ATP availability, or an altered substrates utilization [7, 12].

A shift in energy metabolism from normal using of fatty acids (due to a decline in the expression of genes involved in fatty acid metabolism [13]) towards using glucose, which probably results in differences in substrates oxidation and thus mitochondrial function, has been observed in both ischaemic and nonischaemic heart failure [13–16]. Therefore, with this shift of metabolic profile, the myocardium relies on glycolysis for ATP generation [17, 18]. This situation has important fallouts in specific conditions such as heart failure associated with diabetes [19]. In this case, reduced FA oxidation is not accompanied by an increase in glucose or lactate oxidation to provide ATP, thus causing an energetic deficit in the failing heart that correlates with overall disease severity [13, 18]. Importantly, in diabetes, hyperglycemia per se, independently of FA utilization, is able to lead to cellular derangements and to adaptive and maladaptive processes involving, among many, the renin-angiotensin-aldosterone system, glucose transporters, and AGEs [20, 21]. In this setting, mitochondrial failure to generate enough ATP, coupled to increased ROS generation, with consequent ROS-induced posttranslational modifications of important proteins of the EC coupling machinery is directly involved in diabetic cardiomyopathy [21, 22].

In a failing heart not only ATP synthesis but also ATP storage is altered. Phosphocreatine is ATP storage molecule, which, in presence of ADP, is converted to creatine and ATP by creatine kinase, thereby generating rapidly energy when it is needed. The ratio of concentrations of phosphocreatine to ATP is used as a measure of energy balance. This ratio is found abnormal in heart failure together with the ATP flux [23].

## 5. Oxidative Stress and Heart Failure

**Figure 1:**
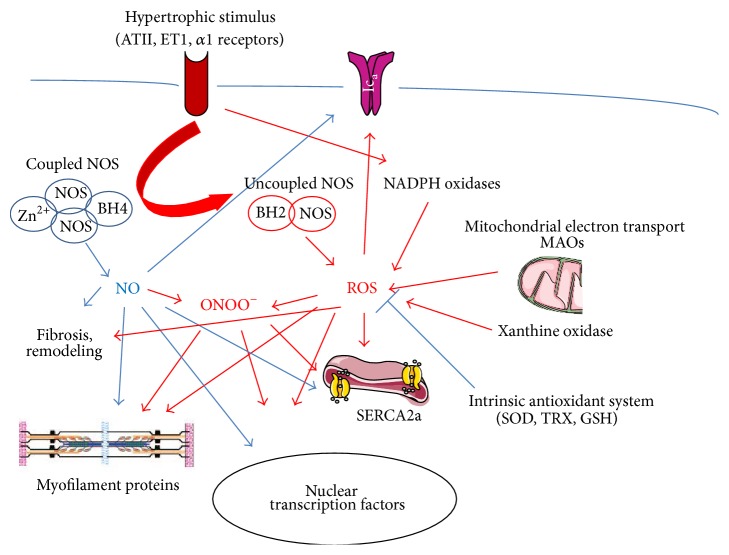
ROS promote heart failure by many mechanisms. Some of the deleterious effects of ROS are evidenced by red arrows. NO counteracts such effects (blue arrows). Modified from Tocchetti et al. [25].

Oxidative stress is commonly referred to as unbalanced ratio between production and scavenging of oxygen radicals with a detrimental oxidation of different substrates (proteins, lipids, nucleic acids, and others). The onset of a prooxidative condition can be due to a hyperactivation of different reactive species sources (see below) or to a depletion of antioxidant defenses or both. It is not clear whether oxidative stress is to be considered a cause, an index, or a mediator of heart failure. In the next paragraphs the sources and effects of prooxidant will be described in order to elucidate the role of oxygen radicals on the etiopathology of a failing heart (Figure 1).

## 6. Sources of Reactive Oxygen and Nitrogen Species in Heart Failure

Both excessive neurohormonal stimulation and energetic deficits with mitochondrial dysfunction lead to increased oxidative stress [21, 24] with production of excessive Reactive Oxygen Species (ROS) and Reactive Nitrogen Species (RNS), widely recognized as promotors of both cardiac dysfunction and pathological remodeling of HF, which is characterized by altered excitation-contraction (EC) coupling with abnormally lower cardiac contractility and muscle relaxation velocities. Among other events linked to HF onset and progression are maladaptive hypertrophic myocardial response, extracellular matrix remodeling, altered tissue energetics, loss of viable cardiomyocytes, vascular and capillary abnormalities, and inflammation [4, 7, 25–30]. Myocardial redox imbalance may be responsible, at least in part, for such abnormalities [22, 31]. ROS and RNS can be produced endogenously by cardiomyocytes by several cellular sources, including “direct” production such as NADPH oxidase system, lipoxygenases, cyclooxygenases, peroxidases, mitochondrial oxidative phosphorylation, nitric-oxide synthase 3 (NOS3 or eNOS) [25–27, 32–34] and “indirect” (free radicals production inducers) such as cytokines, growth factors, angiotensin II, catecholamines, pressure overload, xanthine oxidases, monoamine oxidases, enzymes of catecholamine and serotonin catabolism. Also, the myocardium is provided with endogenous nonenzymatic (i.e., glutathione, vitamins E and C, and *β*-carotene [33], lipoic acid, ubiquinone, and urate) and enzymatic systems that catabolize ROS physiologically generated [25].

NADPH oxidases are important cellular sources of ROS, crucial in many pathophysiological conditions that lead to cardiac diseases [25, 35–37]. The NADPH oxidase enzyme complex is composed of seven catalytic subunits, Nox1–Nox5 and Duox1 and Duox2. Nox2 and Nox4 are expressed in the heart and produce ROS by electron transfer from NADPH to molecular oxygen. In physiological states, Nox2 is quiescent and is stimulated by the translocation of regulatory proteins to activate the oxidase complex on the membrane [25, 38]. G-protein-coupled receptor agonists, cytokines, and growth factors can stimulate Nox2 to generate ROS. On the other hand, Nox4 is constitutively active and is modulated by its expression levels [25, 37]. Both Nox2 and Nox4 are key players in the pathogenesis of LV dysfunction. Indeed, after myocardial infarction, myocytes hypertrophy and apoptosis were significantly reduced in aortic rings of Nox2-deficient mice, with less LV dilation and better function compared to wild type mice [25, 37, 39]. Also, Nox2-containing NADPH oxidases play a role in ATII-induced hypertrophy independently of pressure overload [37]. The underlying mechanism at the base of the prohypertrophic Nox2 effect relies, at least in part, on the oxidation of mitochondrial proteins induced by increased production O_2_^−^ that leads to mitochondrial dysfunction [39]. At the same time, Nox4-containing NADPH oxidases are important in the pathophysiology of cardiac hypertrophy from pressure overload: pressure overloaded hearts from c-Nox4^−/−^ mice showed less hypertrophy and less interstitial fibrosis and apoptosis and had improved LV function compared to wild type [25, 37, 40]. Human failing hearts exhibit increased NADPH oxidase activation [41] together with the parallel activation of downstream signaling components ERK1/2, JNK, and p38 [42]. Also, Nox4 levels increase gradually in aging cardiomyocytes; interestingly, apoptosis is also increased upon enhanced Nox4 expression of caused apoptosis [25, 37]. Nox4 appears to produce mostly H_2_O_2_, while Nox2 generates mostly O_2_^−^ [35, 36].

ROS can be also produced by xanthine oxidase (XO), an enzyme that belongs to the molybdoenzyme family (which comprises enzymes such as aldehyde oxidase and sulfite oxidase) [43]. Both O^2−^ and H_2_O_2_ XO can be generated by oxidative hydroxylation of purine substrates from XO. Inhibition of xanthine oxidoreductase improves cardiac structure and function in spontaneously hypertensive/HF rats [25, 44]. Also, XO can be activated by NAD(P)H oxidase [25, 45]. Compared to wild type animals, myocardial XO activity did not increase after MI in p47phox−/− mice (genetically deprived of p47phox, the cytosolic NADPH oxidase component). Disappointingly, XO inhibitors, such as the purine analog allopurinol and the nonpurine analog febuxostat, when employed clinically, did not exert beneficial effects on human ischaemia/reperfusion and cardiac dysfunction [25], in spite of some success in animal studies [46–48]. Indeed, xanthine oxidase importance and role in the human heart have been questioned [49].

Because of their high-energy needs, cardiac myocytes possess a large number of mitochondria that not only can produce ATP but also can generate ROS as a by-product of mitochondrial respiration. Mitochondrial ROS are produced because the transfer of electrons via the electron transport chain is not totally efficient [50], with O_2_^−^ being generated in the mitochondria at a measurable rate during physiological oxidative phosphorylation. Most of mitochondrial O_2_^−^ possesses a relatively short half-life [43]. In the mitochondria, manganese superoxide dismutase (SOD) is located in matrix while copper/zinc SOD is in the intermembrane space: both of these enzymes can transform O_2_^−^ into H_2_O_2_, that is not so reactive as O_2_^−^ and can easily diffuse and behave as signaling molecule [51]. An alternative enzymatic reaction is operated by other antioxidant enzymes, such as glutathione peroxidase-1 and catalase, which can convert H_2_O_2_ to O_2_ and H_2_O [50]. Nevertheless, an imbalance between mitochondrial prooxidant and antioxidant systems can bring to mitochondrial oxidative stress. Differently from H_2_O_2_, OH (hydroxyl radical) cannot be catabolized by enzymatic reactions but can be quenched only by endogenous or food antioxidants. OH possesses a very short half-life and is very reactive* in vivo*; therefore it is believed to be a very dangerous molecule [52]. OH is a player in reperfusion injury, in HF, stroke, and MI, and in Ca^2+^ cycling and myofilament Ca^2+^ sensitivity in experimental myocardial preparations [33, 53].

Mitochondria produce more ROS during stress conditions, among many ischaemia/reperfusion and cardiac dysfunction [43, 54–56]. ROS can be generated not only on the inner mitochondrial membrane, but also on the outer mitochondrial membrane thanks to monoamine oxidases (MAOs) A and B during oxidative deamination of catecholamines and serotonin [57] (Figure 1).

## 7. Antioxidant Defenses

Antioxidants can be mainly divided into 2 groups: exogenous and endogenous. Antioxidants from exogenous sources are normally introduced with the diet and include (but are not limited to) vitamins (A and C), carotenoids, and flavonoids [58, 59]. On the other hand, endogenous compounds with antioxidant properties can be either of enzymatic origin (superoxide dismutase, GPx, and catalase) or nonenzymatic antioxidants (vitamin E, GSH, and bilirubin) [33]. The therapeutic approach to HF aimed at reducing oxidative stress would benefit from reducing radicals production and by enhancing antioxidant defenses reducing the ratio between the two.

## 8. The Double-Edged Role of Nitric-Oxide Synthases in Cardiac Dysfunction

Nitric-oxide synthases (NOSs) are extremely interesting molecules that produce NO by oxidizing the terminal guanidine nitrogen of L-arginine to L-citrulline. NOSs are present in 3 isoforms [43]: endothelial NOS3 (eNOS) and neuronal NOS1 (nNOS) are constitutively expressed in cardiomyocytes, while inducible NOS2 (iNOS) is absent in the normal myocardium, but its expression can be induced by proinflammatory mediators [25, 60–63]. NO is able to have diverse biological effects by posttranslational nitrosation/nitrosylation of specific cysteine thiol residues [43], mostly due to the cellular location in which NO is generated [28, 64]. NOS3 is mainly located into sarcolemmal caveolae and t tubules, where it interacts with caveolin-3 that modulates its activity and is connected with many cell surface receptors and *β*-adrenergic and bradykinin receptors [43, 65, 66]. NOS3-generated NO has a key role in depressing contractility and regulating *β*-adrenergic stimulation. On the opposite, NOS1 is usually described in the sarcoplasmic reticulum and coimmunoprecipitates with ryanodine receptors (RyRs), thus increasing contractility without altering ICa [25, 28, 61]. Hence, in contrast to NOS3, it appears that NOS1 has mainly a positive inotropic effect on the myocardium [43, 61].

Importantly, NO also plays an essential role in the maintenance of the O_2_^−^/NO homeostasis and can inhibit XO, thus behaving as an antioxidant [25, 67, 68]. In cardiac pathophysiology, maintenance of the nitroso/redox balance between RNS and ROS is critical [25, 27], since excessive oxidative and nitrosative stress are pivotal in many deleterious effects on the myocardium. Indeed, oxidative/nitrosative stress mediate cellular damage to organelles, DNA, proteins, lipids, and other macromolecules and can ultimately bring cardiomyocyte death [29]. Oxidative stress occurs when intrinsic antioxidant defenses are not able to protect from excessive ROS production.

Interestingly, in some pathological conditions, including HF, NOS can be uncoupled, and hence the flow of electrons from the reductase domain to the heme can be diverted to molecular oxygen instead of L-arginine, with following O_2_^−^ production [69, 70]. Among the mechanisms that may be responsible for NOS3 uncoupling, tetrahydrobiopterin (BH4, a fundamental cofactor of NOS) deficiency has been often described [71–73]. Additionally, excessive ROS can further exacerbate NOS uncoupling [25]. NO generated by NOSs is able to react and interact with ROS. Indeed, in HF, ROS and RNS generated by different sources can decrease NO bioavailability. Such interactions can have a significant effect on myocardial contractility [33]. In failing hearts, beyond lower antioxidant defenses, diminished NO levels can bring a further increase in ROS because of NOS uncoupling [64] (Figure 1). Of notice, ROS such as superoxide can directly quench bioavailable NO even without affecting the expression and activity of NOS [74]. Superoxide anion (O_2_^−^) can react with NO, forming reactive species such as peroxynitrite, producing abnormalities in the nitroso-redox balance and further myocardial derangements [26, 30]. Importantly, in cardiomyocytes NO mediates S-nitrosylation of specific cysteines [33, 75], with effects on Ca^2+^ fluxes and EC coupling [33, 76], but high levels of O_2_^−^ can inhibit physiologic S-nitrosylation. High O_2_^−^ concentrations interact with NO to form peroxynitrite that can produce numerous cytotoxic effects that may alter excitation-contraction coupling [26, 77, 78]. Additionally, in failing myocytes, NOS1 moves from its sarcoplasmic reticulum (SR) subcellular location to the sarcolemmal membrane [43, 79], disrupting the tight time- and substrate-dependent NOS regulation. Also, the high levels of NOS2 in failing myocytes appear to be, at least in part, a cause of the blunted myocardial inotropy after *β*-adrenergic stimulation [80, 81].

## 9. ROS-Mediated Alterations in Cardiac Dysfunction

In the heart, ROS stimulate transcription factors to promote hypertrophic signaling, therefore producing cardiac growth, remodeling, and dysfunction. ROS affect cardiac contractility and survival [4, 7, 25–30]. Cardiomyocytes apoptosis that is present in hypertrophy and HF contributes to development and progression of cardiac dysfunction [33, 82]. High levels of ROS have a key role in myocytes apoptosis. Indeed, at relatively low levels, ROS stimulates protein synthesis, while, at higher levels, there is activation of JNK and p38 MAPKs and Akt and induction of apoptosis [33]. Interestingly, in rat cardiomyocytes, H_2_O_2_ at low micromolar concentrations blunts contractile function significantly and activates ERK1/2 kinase with no effect on survival, while at higher micromolar concentrations H_2_O_2_ can stimulate apoptosis via JNK and p38 kinase [52].

ROS mediate the prohypertrophic signaling of alpha 1 adrenergic and angiotensin II pathways [83–87], by means of Ras thiol regulation [88]. In the heart, different signaling pathways involved in the modulation of cardiac hypertrophy, including protein kinase C (PKC), the MAPKs p38, JNK, apoptosis-signaling kinase 1 (ASK-1), and ERK1/2 [33], NF-*κ*B, calcineurin, many tyrosine kinases, Akt, and Phosphatidyl-Inositol-3-Kinase (PI3K) [25–27, 89, 90], can be stimulated by ROS. Interestingly, H_2_O_2_ stimulates hypertrophy by activating PI3K in a time- and dose-dependent manner [91].

ROS can also stimulate myocardial fibrosis, thus contributing to myocardial remodeling [92, 93]: ROS can activate cardiac fibroblasts [94], regulate collagen synthesis [95], and activate posttranslationally matrix metalloproteases (MMPs) that are secreted in an inactive form [96].

Finally, ROS are able to regulate proteins of the excitation-contraction (EC) coupling machinery directly [97] (Figure 1). ROS oxidation of critical thiols on the RyR increases its open probability thus enhancing Ca^2+^ release, exacerbating Ca^2+^ overload and myocyte dysfunction [7, 98, 99]. ROS can also target sarcolemmal L-type Ca^2+^ channel, thus suppressing the Ca^2+^ current [100]. Additionally, they can blunt the activity of the sarcoplasmic reticulum Ca^2+^ ATPase (SERCA2), which plays an essential role in controlling Ca^2+^ cycling, with consequent myocytes dysfunction [33, 101]. Interestingly, low expression of SERCA2a can be already found in myocytes hypertrophied after ROS treatment [33]. Activation of Ca^2+^-calmodulin-dependent protein kinase II (CaMKII) by ROS [102] is critically linked to remodeling of ionic homeostasis in various experimental hypertrophy models [103, 104] including familial hypertrophic cardiomyopathy [105]. In the myocardium, ROS can regulate the function of other important channels, including sodium channels, potassium channels, and ion exchangers, such as the Na^+^/Ca^2+^ exchanger (NCX) and Na^+^/H^+^ exchanger type 1 [33, 106–110]. Also, in HF ROS can contribute to cardiac dysfunction by lowering myofilaments Ca^2+^ sensitivity [111, 112].

## 10. Antioxidant Therapeutics in Heart Failure

### 10.1. Standard Heart Failure Therapy That Possess Antioxidant Properties

During the last decades, treatment of HF has changed more than one time, along with the progressing pathophysiological knowledge of the disease. It initially focused on hemodynamic control and unloading of the heart with vasodilators and diuretics. Then, the concept that the compensatory neurohormonal response was no longer considered beneficial but rather worsening heart failure introduced inhibitors of renin-angiotensin-aldosterone system, as well as *β*-blockers, now used as current therapeutics. Then treatments focused on muscle stimulation in the weakened heart, but these therapeutics were set aside due to their detrimental effects when used in the long term [113] as demonstrated by several clinical trials [1, 4, 7, 114–118]. In the last years, implantable devices have had a remarkable impact on management of heart failure, since electrical devices controlled by microprocessors can deliver therapy, monitor disease, and prevent sudden cardiac death [2, 3, 7]. Interestingly, it has to be acknowledged that standard HF therapy is based on many drugs that possess redox properties (Table 1) [43]. For instance, current treatments with ACE inhibitors and ATII receptor blockers (ARBs) can limit ROS deleterious signaling [43, 119]. Indeed, ATII can induce hypertrophy via a G*α*q mediated pathway that involves ROS generation and ROS-associated activation of various downstream signals [85, 120]. Consequently, in clinical practice, blockade of either ATII production or ATII binding to the AT1 receptor can prolong survival in patients. Notably, antioxidants that counteract ROS effects can also blunt ATII-induced hypertrophy [43, 86].

Spironolactone inhibits aldosterone actions, blunting the myocardial oxidant and profibrotic conditions that are a hallmark of HF. Indeed, aldosterone is able to activate NADPH oxidases, thus increasing ROS production [43, 121]. Similarly, correction of redox imbalance has been implicated in the therapeutic effects of eplerenone in HF [43, 122, 123].

Interestingly, recent studies on cardiac resynchronization therapy (CRT) demonstrate that the beneficial effects of this important device therapy also involve, among many, a redox-mechanism. In particular, in dyssynchronous HF, Cys294 of the mitochondrial F1-ATPase can form a disulfide bond with another cysteine residue, while introduction of CRT prevents disulfide formation with S-nitrosylation of Cys294 [124, 125].

Carvedilol is *β*1- and *β*2-adrenergic receptor antagonist with additional vasodilatory *α*1-blocking properties [126]. Its structure contains a carbazole moiety by which carvedilol can be considered also a potent antioxidant [126–128], as a result of increased NO production or decreased inactivation [126, 129].

The third-generation *β*-blocker nebivolol, by simultaneous stimulation of *β*3-adrenergic receptor (AR), can enhance NO signaling which is often lost in HF because of the lower NO bioavailability. The eNOS-dependency of nebivolol beneficial effects beyond conventional beta blockers was demonstrated in experimental models of post-MI and hypertrophy [130, 131]. A recent study [132] also showed that microdomain-targeted enhancement of myocardial *β*3AR/NO-cGMP signaling may be responsible, at least in part, for *β*1-adrenergic antagonist-mediated preservation of cardiac function in a volume-overloaded canine model. Additionally, the BEAT-HF trial (NCT01876433) is recently evaluating efficacy of oral treatment with a *β*3AR agonist in chronic HF, exploring also potential effects on diastolic function, symptoms, repolarization duration, and safety (Table 1).

### 10.2. Drugs with Redox Effect That Are Not Mainstream Therapeutic Approach to Heart Failure

Potentiating NO/cGMP signaling has provided beneficial effects on animal models of HF by means of phosphodiesterases 5 (PDE5) inhibition [133] and by BH4 supplementation [73]. cGMP/PKG (cGMP-dependent protein kinase) pathway negatively controls stress-response signaling. cGMP is generated upon natriuretic peptide binding to its receptors coupled to particulate guanylyl cyclase or upon NO activation of soluble guanylyl cyclase. Importantly, cGMP controls the activities of phosphodiesterases (which in turn control cAMP and cGMP hydrolysis) and can then activate PKG. This important kinase phosphorylates Ca^2+^ channels, myosin phosphatase, RGS2 (which negatively regulates G-protein-coupled receptors), and IRAG (which modulates inositol-1,4,5-trisphosphate-dependent Ca^2+^ signaling), troponin I, and phospholamban [134]. Enhancing cGMP/PKG signaling by inhibiting PDE5 seems to be able to attenuate and reverse cardiac hypertrophy induced by pressure overload [133] and blunt acute and chronic *β*-adrenergic stimulation and also protect against ischaemia-reperfusion injury and myocardial apoptosis induced by antitumoral agents [135–137]. Even though the first clinical trials with sildenafil in HF have been somehow disappointing, the concept that the cGMP pathway is a promising target to exploit has been corroborated by the recent results on the beneficial effects of neprilysin inhibition combined to ARBs [138].

Simvastatin (NADPH oxidase inhibitor) and allopurinol (xanthine oxidases inhibitor) both counteract oxidative stress and interfere with ROS-mediated hypertrophic signaling [139], blunting cardiac remodeling. In particular, statins can inhibit the isoprenylation and activation of Rac1 and other proteins of the Rho family, hence lowering NADPH oxidase activity [43, 140]. Additionally, it seems that statins have direct antioxidant effects on lipids, and it has been shown that the oxidation of LDL, VLDL, and HDL can be inhibited by hydroxyl metabolites of atorvastatin [43, 141]. Also, both short- and long-term therapies with statins can benefit endothelial dysfunction [43]. Recent work from Andres and colleagues [142] showed that acute cardioprotective effects elicited by simvastatin involve the protein Parkin that stimulates mitophagy and prevents mevalonate accumulation. The xanthine oxidase inhibitor allopurinol is currently studied to improve remodeling after MI in diabetic patients (clinicaltrials.gov: NCT01052272) [139].

Enhanced myocytes [Na^+^]_i_ has been recently shown to lower mitochondrial Ca^2+^ uptake, increasing ROS production [110]. The same group was able to prevent such enhanced ROS generation with an inhibitor of the mitochondrial Na^+^/Ca^2+^ exchanger (mNCE), which decreased Na^+^-induced Ca^2+^ exportation [109]. In turn, ROS could then activate Ca^2+^/calmoduline kinase II [104, 143] that would increase late I_Na_ by interacting with the Na^+^ channel [144, 145], thus generating a vicious cycle of high [Na^+^]_i_ and oxidative stress [110]. High [Na^+^]_i_ would then stimulate NCX and intracellular Na^+^ would be exchanged with extracellular Ca^2+^ with consequent Ca^2+^ overload and electrical and mechanical dysfunction, in a scenario in which SERCA2a is inhibited and the RyR2 is activated by ROS [146, 147]. Hence, high [Na^+^]_i_ can be identified as an interesting therapeutic target for HF treatment [102]. Indeed, inhibiting the late I_Na_ with ranolazine has been proven beneficial in experimental HF [102, 148–151].

Other promising therapeutic targets are monoaminoxidases: MAO A and MAO B have been recently proposed to play a role in experimental hypertrophy and failure via increased generation of H_2_O_2_. Pharmacological or genetic manipulation of such enzymes could then prove beneficial in cardiac dysfunction [25, 152, 153] (Table 1).

### 10.3. Novel Therapeutic Compounds That Target the ROS/RNS Signaling Pathways

Other interesting compounds that may ameliorate cardiac function by acting on the redox milieu have been identified. SS-31 (MTP-131, Bendavia) [154] is a mitochondria-specific antioxidant that appears to decrease LV hypertrophy in a mouse model of ATII-induced hypertrophy [155] and improve postinfarction cardiac function preventing adverse left ventricular remodeling and restoring mitochondria-related gene expression in rats [156]. Four phase I trials with Bendavia have been completed, with an ongoing phase II trial in ischaemic cardiomyopathy [139, 157].

Resveratrol is a widely used antioxidant dietary supplement with promising experimental results on pressure overload cardiac hypertrophy, but beneficial effects on clinical hypertrophy have not yet been reported [139, 158].

Currently, in HF treatment the room for inotropic therapies such as dopamine, dobutamine, and milrinone is very limited by the mortality associated with long-term treatment with these drugs [115–117]. Nitroxyl (HNO) represents an alternative approach. HNO is a 1-electron-reduced and protonated sibling of NO and, like NO, is a gaseous signaling molecule and a potent vasodilator. Nevertheless, HNO appears to have distinct chemical and physiological properties and unique signaling pathways from those of NO [159, 160]. HNO was initially discovered to induce both venous and arterial dilation and positive inotropy in intact failing hearts. Following mechanistic studies have revealed multiple pathways that combine the strategies of these other approaches. Clinical interest in HNO is increasing in virtue of its positive inotropic effects. In vitro experiments suggested positive inotropic and lusitropic properties of HNO, while subsequent studies in healthy and heart failure dog models with the HNO donor Angeli's salt (Na_2_N_2_O_3_) demonstrated significant improvements in load-independent LV contractility, associated with reductions in preload volume and diastolic pressure [161, 162]. These beneficial effects seem to be independent of cAMP/protein kinase A (PKA) and cGMP/PKG signaling [163] with no modification of L-type calcium channel activity [164], but rather related to modifications of specific cysteine residues on phospholamban [165, 166] and SERCA2a [167] and on myofilament proteins, correlating with increased Ca^2+^ sensitivity and force generation [168]. Recently, a new HNO donor, CXL-1020, has been developed, and both animal and clinical studies seem to confirm positive inotropic and lusitropic effects [118, 169–171] (Table 1).

## 11. Conclusions

ROS and RNS at physiological concentrations are beneficial molecules and play a role in the regulation of cellular signaling pathways [28]. ROS/RNS generation is finely regulated for proper myocardial homeostasis. Although oxidative and nitrosative stress can be deleterious and may therefore constitute a therapeutic target in HF, indiscriminate elimination of ROS and RNS by antioxidant treatments may not provide any improvement and may even impair physiological cellular functions, causing a complete loss of ROS/RNS signaling [172–175]. Indeed, antioxidants were shown to be able to counteract cardiac remodeling and improve contractility in many animal models of HF. However, when translated to the clinical arena, such therapeutic strategies [64] did not show the expected benefits or even worsened mortality [176], when the antioxidant effect was not paralleled by other pharmaceutical and biological properties, as for carvedilol [126]. Importantly, ROS biological effects on cardiomyocytes depend on the site of generation. Therefore, more specific, targeted, and “compartmentalized” antioxidant approaches that blunt local ROS/RNS production might be more successful in countering irreversible oxidative modifications. Furthermore, since in heart disease deranged mitochondria are the major generators of ROS, dictating the overall myocardial redox conditions, therapeutic strategies aimed at removing diseased mitochondria, thus promoting mitophagy, may help diminishing oxidative stress and ameliorating cardiac function [176].

## Figures and Tables

**Table 1:** Properties of the main antioxidant therapeutics.

*Components of standard heart failure therapy that possess antioxidant properties*	
ACEi, ARBs, ARNi, antialdosterone drugs: interference with RAAS signaling	
Carvedilol: *β*1- and *β*2-adrenergic receptor blocker that also increase NO production or decrease inactivation	
*β*3AR agonists: enhancement of myocardial *β*3-adrenergic coupling with NO-cGMP signaling	
ARNi: enhancement of NPs/cGMP/PKG pathway	

*Drugs with redox effect that are not mainstream therapeutic approach in heart failure*	
PDE5 inhibition and BH4 supplementation: potentiating NO/cGMP/PKG signaling	
Statins: NADPH oxidase inhibitors	
Allopurinol: xanthine oxidases inhibitor	
Ranolazine: inhibitor of elevated late I_Na_	
MAO inhibitors: blunting ROS production from MAOs	

*Novel therapeutic compounds that target ROS/RNS signaling pathways*	
SS-31 (MTP-131, Bendavia): direct action on mitochondrial function	
Resveratrol: preservation of the LKB1-AMPK-eNOS signaling axis	
HNO donors: improving Ca^2+^ cycling and myofilament Ca^2+^ sensitivity	

ARNi: angiotensin receptor-neprilysin inhibitor.

AMPK: AMP-activated protein kinase.

NPs: natriuretic peptides.
